# Ultrastructural Characterization of PBMCs and Extracellular Vesicles in Multiple Sclerosis: A Pilot Study

**DOI:** 10.3390/ijms25136867

**Published:** 2024-06-22

**Authors:** Roberto De Masi, Stefania Orlando, Elisabetta Carata, Elisa Panzarini

**Affiliations:** 1 Complex Operative Unit of Neurology, “F. Ferrari” Hospital, Casarano, 73042 Lecce, Italy; 2 Laboratory of Neuroproteomics, Multiple Sclerosis Centre, “F. Ferrari” Hospital, Casarano, 73042 Lecce, Italy; 3 Department of Biological and Environmental Sciences and Technologies (Di.S.Te.B.A.), University of the Salento, 73100 Lecce, Italy; elisa.panzarini@unisalento.it

**Keywords:** multiple sclerosis, PBMCs, electron microscopy, extracellular vesicles

## Abstract

Growing evidence identifies extracellular vesicles (EVs) as important cell-to-cell signal transducers in autoimmune disorders, including multiple sclerosis (MS). If the etiology of MS still remains unknown, its molecular physiology has been well studied, indicating peripheral blood mononuclear cells (PBMCs) as the main pathologically relevant contributors to the disease and to neuroinflammation. Recently, several studies have suggested the involvement of EVs as key mediators of neuroimmune crosstalk in central nervous system (CNS) autoimmunity. To assess the role of EVs in MS, we applied electron microscopy (EM) techniques and Western blot analysis to study the morphology and content of plasma-derived EVs as well as the ultrastructure of PBMCs, considering four MS patients and four healthy controls. Through its exploratory nature, our study was able to detect significant differences between groups. Pseudopods and large vesicles were more numerous at the plasmalemma interface of cases, as were endoplasmic vesicles, resulting in an activated aspect of the PBMCs. Moreover, PBMCs from MS patients also showed an increased number of multivesicular bodies within the cytoplasm and amorphous material around the vesicles. In addition, we observed a high number of plasma-membrane-covered extensions, with multiple associated large vesicles and numerous autophagosomal vacuoles containing undigested cytoplasmic material. Finally, the study of EV cargo evidenced a number of dysregulated molecules in MS patients, including GANAB, IFI35, Cortactin, Septin 2, Cofilin 1, and ARHGDIA, that serve as inflammatory signals in a context of altered vesicular dynamics. We concluded that EM coupled with Western blot analysis applied to PBMCs and vesiculation can enhance our knowledge in the physiopathology of MS.

## 1. Introduction

Vesiculation is a constitutive process of cellular membranes, although it can also be modulated by external physicochemical factors. Nanometer-sized extracellular vesicles (EVs) are ubiquitously produced by both prokaryotic and eukaryotic cells, and they are involved in physiological and pathological processes. EVs are currently considered key players in intercellular signaling, since they can travel long distances and deliver their messages to the acceptor cells. Depending on the origin cells, these messages correspond to several molecules, including lipids, proteins, and nucleic acids, as well as having different biological significance, resulting in functional modifications of target cells, for example, in both pro-inflammatory and anti-inflammatory senses. Consistently, growing evidence indicates that EVs are important players in autoimmune disorders, including multiple sclerosis (MS), a chronic autoimmune disease [1,2,3]. In spite of their emerging importance in the physiology of cells and organs, these structures are currently underexplored. This also applies to demyelinating diseases and MS. MS is a chronic demyelinating autoimmune disease of the central nervous system (CNS) with a degenerative and inflammatory component. Although its etiology remains unknown, its molecular physiology has been well studied, indicating the peripheral blood mononuclear cells (PBMCs) as the main pathologically relevant contributors in the disease and immuno-mediated neuroinflammation. Specifically, the degenerative component is thought to be secondary or primary, according to the outside-in or the inside-out theory [4]. In any case, neuroinflammation takes place as the driving pathological process and the inflammatory demyelination represents a common mechanism, beginning with the breakthrough of peripheral immuno-tolerance. It is commonly accepted that antigen presenting cells (APCs) can have a pathogenic role in MS when they express a foreign oligopeptide epitope. In the presence of this epitope, T cell receptors, HLA MHCII restricted pockets, and APCs trigger the trimolecular complex. The latter, in turn, can constitute an immunological trigger, depending on matching similarities between self-structures and the presented epitope, resulting in molecular mimicry. Finally, this phenomenon can induce T cell peripheral activation and self-reactive clonal expansion [5]. The enhancement of this biological cascade occurs if there is a concomitant imbalance between proinflammatory (M1) and immunomodulatory (M2) monocytes (the so called M1/M2 imbalance) [6]. This causes the breakdown of the blood–brain barrier (BBB) and consequent invasion of the CNS by peripheral blood-derived lymphocytes as well as monocytes and their reactivation process, resulting in focal inflammation, axonal loss, and clinical relapse [7]. Subsequent relapses could derive from this process due to epitope spreading into the CNS, and so on for the other demyelination waves. Stable neurological impairment and residual disability accumulation after each relapse are correlated with axonal loss, rather than inflammation. In fact, neuroinflammation and neurodegeneration are considered interacting and colocalized aspects of the same pathological process [8].

In the last two decades, it has been possible to partially prevent both of these processes by using approved disease modifying treatments (DMTs), such as Interferon (IFN) β-1a and β-1b [9]. They cannot penetrate the BBB but can modulate the immune system in the periphery. Thus, IFN-induced reduction in the annual relapse rate and lesion load stabilization are obtained only by acting on the PBMCs, confirming them as key players in MS pathophysiology. The phenotypic composition of PBMCs is very heterogeneous as is their pathological modulation. The latter is known to be subjected to a cascade mechanism involving selectins, cytokines, and, finally, integrins; however, the intimate cell-signaling pathway is still largely elusive. For this purpose, membrane trafficking and EV release are thought to be important inflammatory modulators, since EVs can cross the BBB in both directions, transducing cell-to-cell communication signals both from the periphery to CNS and vice versa. Consistently, EVs have garnered a growing interest in neurological diseases as carriers of pivotal molecules, including nucleic acids, proteins, lipids, and metabolites, and also as potential biomarkers, since they remotely reflect molecular events occurring in the brain [10].

According to the Minimal Information for Studies of Extracellular Vesicles (MISEV) guidelines, proposed by the International Society for Extracellular Vesicles (ISEV), EVs can be categorized based on (a) physical characteristics, such as size (small EVs, sEVs and large EVs, *l*EVs, with ranges defined <200 nm, or >200 nm, respectively) or density; (b) biochemical composition; (c) descriptions of conditions or cell of origin, or biogenesis mechanisms (endosomal compartment or plasma membrane budding). In the case of the latter, EVs originating from the endosomal compartment are defined as exosomes (EXOs, 30–200 nm), formed by exocytosis of intracellular vesicles called multivesicular bodies (MVBs), while EVs budding from the plasma membrane are known as microvesicles (MVs, up to 1 μm). Finally, the apoptotic bodies (ApoBDs, 1–5 μm) are a product of apoptosis and contain the biomaterial from dying cells [11,12].

In particular, both EV types originating from the endosomal compartment and those budding from the plasma membrane are known to be involved in CNS physiological homeostasis as well as in neuroinflammation [13].

Recently, our group postulated a pathogenesis of MS based on the protein misfolding/unfolding process and consequent ER stress. This postulate was sustained by the observation of dysregulation in PBMC GANAB expression, the main enzyme involved in the protein maturation process [14,15]. However, the expected effect of protein misfolding/unfolding on membrane trafficking is still to be confirmed in MS. In fact, putative intracellular accumulation of unfolded or misfolded proteins can affect the endosomal compartment and EV budding as a result of membrane trafficking dysregulation. Thus, the EV/PBMC axis can be considered of great interest due to its pathogenic relevance in MS, but also as a critical player in neuroinflammation and consequent neurodegeneration. Nevertheless, the role of peripheral immunity and its interaction with CNS immunity and EVs is still in the beginning phase and requires adequate methodological tools to be investigated.

Electron microscopy (EM) has few applications in demyelinating diseases, and related findings concern the study models, such as experimental allergic encephalitis (EAE) [16]. Thus, no published papers studying PBMCs in MS, or related disorders, are present in the EM field. EM allows for the discrimination of details occurring at the cellular and subcellular levels, enabling researchers to visualize cellular structures, organelle dynamics, and molecular interactions. Moreover, the coupling of ultrastructural analyses with omics technologies, such as proteomics, can bridge the gap existing between structural modifications and molecular processes, offering a deeper, integrated morpho-functional approach to the study.

Here, we aim to primarily evaluate the applicability of EM coupled with Western blotting analysis, in order to investigate PBMCs and EVs in MS patients, and to understand if ultrastructural alterations in PBMCs are related to the pathology. To this purpose, we evaluated the ultrastructural morphology of PBMCs, the type of EVs present in the blood plasma of MS patients, and the content of these vesicles. In particular, we assessed the presence in EVs of GANAB, Cortactin, IFI35, TGFβRII, NF-κB, Septin 2, Cofilin 1, and ARHGDIA, which serve as inflammatory signals in a context of altered vesicular dynamics and altered membrane trafficking.

## 2. Results

The mean age of HCs and RRMS patients was 41.2 *±* 3 (min 39, max 43) years and 39.8 *±* 2 (min 38, max 42) years, respectively. Patients had DD and AO of 13.3 years (min 8.2, max 14.4) and 28.1 (min 26, max 32.0) years, respectively. As regards the age, we noted no difference between HCs and MS patients (*p* = 0.51).

### 2.1. TEM

PBMCs are a heterogeneous cell population including monocytes (which can differentiate into macrophages and dendritic cells) and lymphocytes (T cells, natural killer cells, and B cells). PBMCs can also contain platelets as well as erythrocytes and granulocyte contamination, depending on the laboratory extraction procedure.

**Figure 1:**
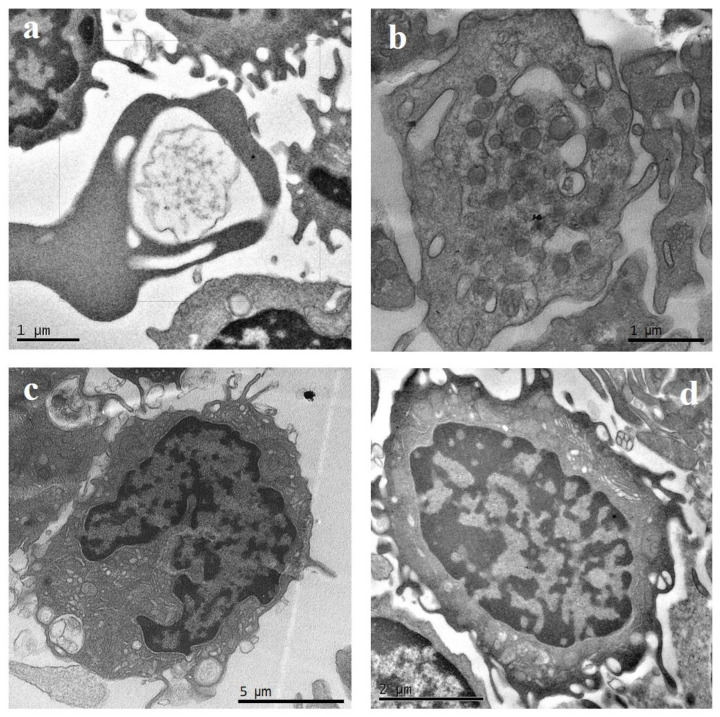
Transmission electron microscopy (TEM) representative images of peripheral blood mononuclear cells (PBMCs) from a healthy control. (**a**) Erythrocytes; (**b**) platelets; (**c**) monocytes; (**d**) lymphocytes.

Figure 1 shows the TEM of PBMCs from a healthy control. From the cytological point of view, in HCs, inactivated and resting state cells were observed. In particular, the presence of a nucleus allowed us to distinguish immune cells from erythrocytes (Figure 1a) and platelets (Figure 1b), while morphology distinguishes PBMCs from granulocytes, characterized by a lobulated nucleus as well as by cytoplasmic granules. Monocytes had a diameter ranging between 12 and 20 μm, with a kidney- or bean-shaped nucleus displaying dispersed chromatin and plasma membrane with pseudopod-like extensions (Figure 1c). Lymphocytes had a diameter ranging between 5 and 10 μm, with a central nucleus, a thin cytoplasm, and a smooth plasma membrane with fewer projections than in samples of disease subjects (Figure 1d).

**Figure 2:**
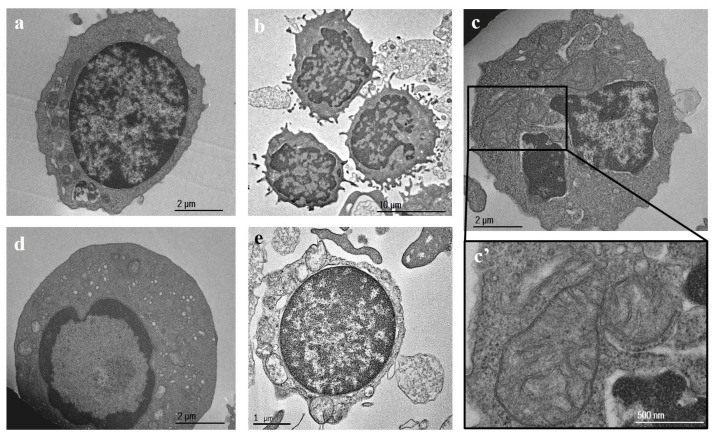
Transmission electron microscopy (TEM) representative images of peripheral blood mononuclear cells (PBMCs) from an MS patient. (**a**,**b**) Lymphocytes; (**c**,**c’**) mitochondria; (**d**) apoptotic lymphocyte; (**e**) necrotic lymphocyte. Note the plasmalemmal pseudopods and the damaged mitochondria (indicated by loss or swelling of cristae).

In MS patient samples, the number of activated lymphocytes displayed an increased plasma membrane ruffling and abundant cytoplasm compared to controls, and more indented and/or convoluted nuclei (Figure 2a,b) were observed; apoptotic cells showed chromatin condensation by budding (Figure 2d), or necrotic cells (Figure 2e) were present. Mitochondria showed evidence of damage, as indicated by condensation and loss or swelling of cristae, which may be indicative of primary or secondary mitochondrial dysfunction (Figure 2c,c’).

**Figure 3:**
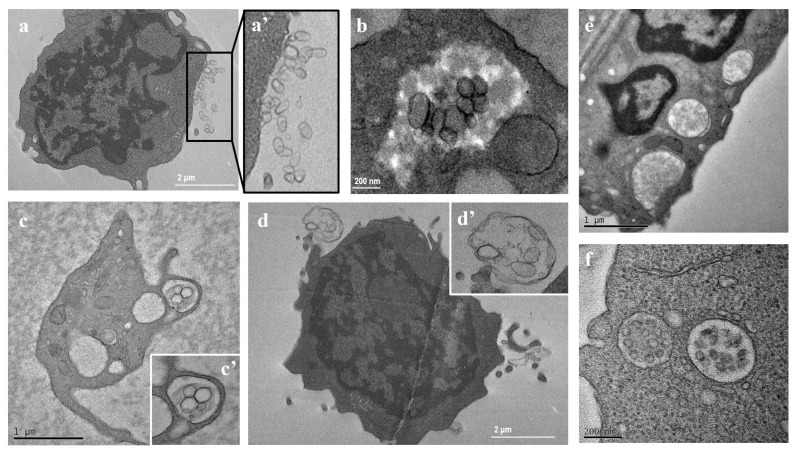
Transmission electron microscopy (TEM) representative images of peripheral blood mononuclear cells (PBMCs) from MS patients. (**a**,**a’**) Vesicles released by PBMCs; (**b**) vesicles within cytoplasm; (**c**,**c’**,**d**,**d’**) membrane-surrounded structures; (**e**) autophagosomes; (**f**) multivesicular bodies. Note the number of cytoplasmic multivesicular bodies, large vesicles budding from the plasmalemma, and, in some cases, the amorphous material surrounding the vesicles present within the cytoplasm.

Interestingly, PBMCs from MS patients also showed an increment in the number of multivesicular bodies (MVBs) within the cytoplasm. MVBs contained vesicles that are released from cells as single vesicles (Figure 3a,a’) or as vesicles contained within membrane-surrounded structures (Figure 3c,c’,d,d’,f). In some cases, amorphous material was observed around the vesicles present within the cytoplasm (Figure 3b). In addition, a high number of plasma-membrane-covered extensions, with multiple large vesicles associated and numerous autophagosomal vacuoles containing undigested cytoplasmic material, were observed (Figure 3e).

Finally, we calculated the number of MVBs from HCs, resulting in an average of 15.5 ± 3.1/µ^3^. Likewise, we calculated the number of MVBs from MS patients, resulting in an average of 29.2 ± 2.8/µ^3^, with a statistical difference in comparison to those of HCs (*p* = 0.0012). This evaluation was made taking into account all images of each studied subject.

### 2.2. EV Characterization

**Figure 4:**
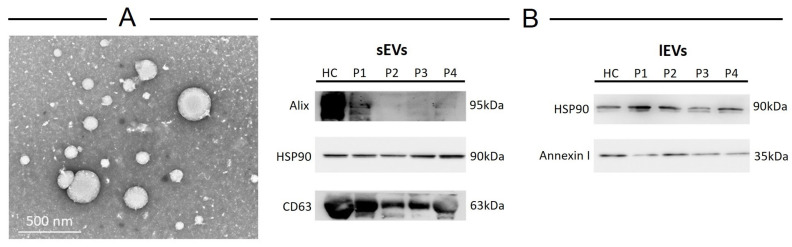
Characterization of large and small extracellular vesicles isolated from plasma of healthy controls and MS patients. (**A**) Transmission electron microscopy of vesicles; (**B**) Western blotting of proteins isolated from vesicles.

MS patients’ blood was collected at the time of clinical consultation and processed as detailed in the methods section. Vesicles were isolated from 5 mL of plasma by ultracentrifugation and analyzed by transmission electron microscopy to evaluate size and morphology (Figure 4A). We observed two types of vesicle populations: one (84% of observed vesicles) having an average diameter of 87 nm and the other (16% of observed vesicles) with an average diameter of 240 nm, thus belonging to the sEV and *l*EV sub-groups of EVs, respectively. In addition, there was no significant difference in the mean size of particles between HCs and MS patients. Independently of diameter, the vesicles have a spherical morphology; conversely, only bigger vesicles show electron-dense material around the membrane. Western blotting of protein extracts for characteristic extracellular vesicles (i.e., CD63, Alix, Annexin I, Tsg101, Flotilin, Calnexin, HSP90) showed that only the endosomal marker CD63 and Alix, as well as the plasma membrane protein Annexin I, were expressed in vesicles isolated from MS patients; moreover, in both small and large fractions HSP90 was detected (Figure 4B). Finally, both EV fractions were negative for calnexin, an endoplasmic reticulum resident protein generally absent in EVs, confirming the validity of the chosen isolation technique.

GANAB, TGFβRII, NF-κB, Cortactin, Septin 2, IFI35, ARHGDIA, and Cofilin 1 abundance in EVs isolated from the peripheral blood of healthy and MS patients was determined by Western blotting, the densitometric analysis highlighting a significant difference between HC- and MS-patient-derived EVs as well as between the *l*EVs and the sEVs.

In general, a significant high quali-quantitative variability in the content of vesicles isolated from MS patients was observed, with some differentially expressed characterizations: (I) ARHGDIA and Cofilin 1 were present only in the small vesicles; (II) patient 3 displayed the highest amount of considered proteins from the large vesicles, except for IFI35 which is more expressed in the small vesicles; (III) in the small vesicles from HCs, the molecules were less abundant than in large ones (Table 1).

Furthermore, in vesicles isolated from healthy controls, the proteins considered in this study were more abundant in large vesicles rather than in small ones, with a gradient in the amount differences, from TGFβRII to IFI35 (in particular, TGFβRII > Cortactin > NF-κB > Septin 2 > GANAB > IFI35 for *l*EVs and Cofilin 1 > NF-κB > Septin 2 > TGFβRII > GANAB > Cortactin > IFI35 for sEVs).

The corresponding arbitrary densitometric unit percentages (ADUs) in large vesicles were 36.62 ± 1.93, 33.50 ± 1.77, 28.60 ± 1.39, 22.38 ± 0.99,17.14 ± 0.86, 14.79 ± 0.7 for TGFβRII, Cortactin, NF-κB, Septin 2, GANAB, and IFI35, respectively, while the ADUs in small vesicles were 17.95 ± 0.87, 9.57 ± 0.47, 7.13 ± 0.36, 6.91 ± 0.27, 5.51 ± 0.28, 0.54 ± 0.03, 0.04 ± 0.01 for Cofilin 1, NF-κB, TGFβRII, Septin 2, GANAB, Cortactin, and IFI35, respectively (Table 1).

As regards the single patients, interesting findings were observed too. Specifically, in patient 1 the molecules were more abundant in sEVs, except for Cortactin which showed no significant difference in the amount measured in small and large vesicles. In this compartment, we noted an expression gradient between molecules as follows: GANAB (36.58 ± 1.6) > TGFβRII (29.10 ± 1.44) > IFI35 (27.76 ± 1.38) > Cofilin 1 (24.01 ± 1.1) > NF-κB (23.29 ± 1.1) > Septin 2 (18.4 ± 0.94) > ARHGDIA (12.41 ± 0.65) > Cortactin (8.13 ± 0.39). In the large vesicles, the expression gradient of molecules was GANAB > IFI35 > Cortactin > NF-κB according to the corresponding ADU values of 18.74 ± 0.98, 14.17 ± 0.72, 11.06 ± 0.53, and 0.88 ± 0.04, respectively (Table 1).

In patient 2, the ADU of Septin 2 was the highest in small vesicles (33.17 ± 1.53 in sEVs vs. 23.22 ± 1.13 in *l*EVs), whereas IFI35 was more abundant in *l*EVs (26.98 ± 1.32 in *l*EVs vs. 0.05 ± 0.01 in sEVs). ARHGDIA was absent both in small vesicles and in large ones. NF-κB was comparable between sEVs and *l*EVs (24.10 ± 1.2 and 25.66 ± 1.3, respectively). TGFβRII, Cortactin, and GANAB were more abundant in sEVs compared to *l*EVs (26.97 ± 1.36 vs. 0.35 ± 0.01; 21.17 ± 1.01 vs. 12.15 ± 0.56; 10.04 ± 0.47 vs. 2.81 ± 0.18, respectively). Cofilin 1 of this subject had the lowest ADU within the patient group (14.74 ± 0.77).

As stated above, the EVs isolated from patient 3 evidenced the highest number of proteins in the content of *l*EVs compared to sEVs. This was not true only for the IFI35, which, instead, was more expressed in the small vesicles (59.81 ± 2.88 vs. 29.2 ± 1.52 in *l*EVs). The ADU of ARHGDIA from the sEVs was the highest measured in all EVs (57.92 ± 2.76). The other relative ADUs measured were 55.96 ± 2.8, 38.23 ± 1.91, 37.19 ± 1.85, 37.18 ± 1.91, and 16.07 ± 0.73 for TGFβRII, Septin 2, Cortactin, NF-κB, and GANAB, respectively, in *l*EVs; and 22.36 ± 1.23, 21.13 ± 0.99, 20.81 ± 0.96, 20.49 ± 0.98, 19.41 ± 1.01, and 11.62 ± 0.5 for Septin 2, NF-κB, Cofilin 1, TGFβRII, Cortactin, and GANAB, respectively, in sEVs (Table 1).

Finally, in the EVs isolated from patient 4, we measured the highest GANAB of 45.24 ± 1.99 in *l*EVs (36.26 ± 1.77 in sEVs) and Cortactin of 51.24 ± 2.6 in sEVs (6.1 ± 0.28 in *l*EVs). The ADU of Septin 2 was comparable between sEVs and *l*EVs (18.94 ± 0.97 vs. 16.18 ± 0.76, respectively) and IFI35 (12.34 ± 0.77 vs. 14.86 ± 0.7, respectively). The amount of TGFβRII (7.06 ± 0.29) and NF-κB (7.67 ± 0.38) measured in *l*EVs compared to sEVs (16.54 ± 0.79 and 21.90 ± 1.05, respectively) was lower. The relative ADUs of ARHGDIA and Cofilin 1 in sEVs were 40.84 ± 2.1 and 22.49 ± 1.15, respectively (Table 1).

**Figure 5:**
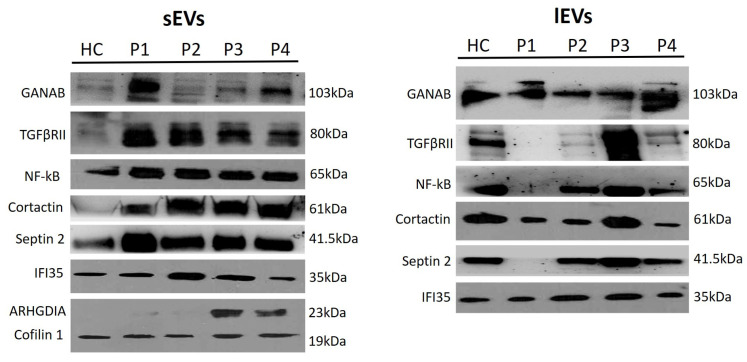
Representative image of Western blotting membranes of characterization of cargo of small (sEVs) and large (*l*EVs) vesicles isolated from the plasma of healthy controls and MS patients.

**Figure 6:**
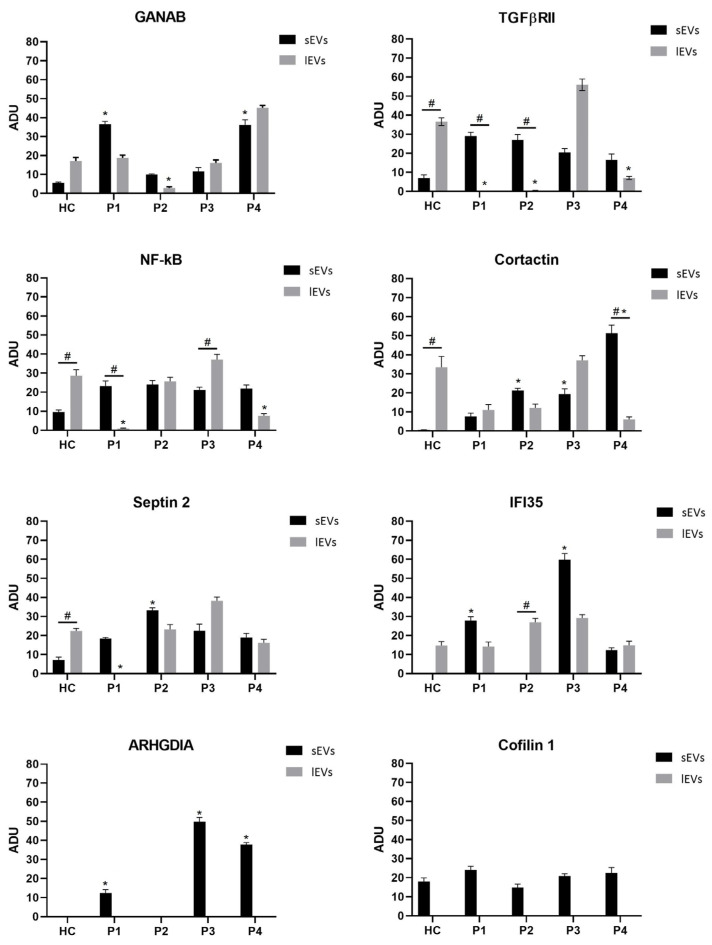
The expression levels of GANAB, TGFβRII, NF-κB, Cortactin, Septin 2, IFI35, ARHGDIA, and Cofilin 1 in large (*l*EVs) and small (sEVs) vesicles isolated from the plasma of healthy controls (HC) and MS patients (P1–P4) evaluated by Western blotting analysis. The expression of these molecules was quantified by densitometric analysis performed by ImageJ (ImageJ 1.53e, Wayne Rasband and contributors, National Institutes of Health, USA). The relative density of the analyzed proteins was calculated by dividing the corresponding peak area by those of the control band from the same sample and reported as arbitrary densitometric units (ADUs). The values for all experimental groups reported in the histograms represent the means ± SD (n = 3) of three independent evaluations (three gels on one sample). Furthermore, each line from P1–P4 is derived from the corresponding individually loaded patient; the line from HC is derived from a sample made up of a pool of the four control subjects. (*) *p* < 0.05 compared to healthy control (HC); (#) *p* < 0.05 comparison between to two EV fractions (sEVs and *l*EVs). Note the expression of ARHGDIA and Cofilin 1 only in sEV compartments.

Figure 5 shows a representative image of Western blotting membranes of characterization of cargo of large (*l*EVs) and small (sEVs) vesicles isolated from the plasma of HCs and MS patients. Figure 6 shows sEV and *l*EV cargo expression of each molecular studied factor, in terms of ADU, in MS patients and healthy controls.

## 3. Discussion

Multiple sclerosis (MS) is characterized by chronic inflammatory demyelination of the CNS and optic nerves with a degenerative component. The occurrence of the disease relies on the interaction between genetic susceptibility and environmental risk factors [17,18], constituting the main cause of non-traumatic disability in young adults [19]. MS presents different disease phenotypes, including relapsing-remitting MS (RRMS), secondary progressive MS (SPMS), and primary progressive MS (PPMS), displayed by approximately 85%, 50–90%, and 10–15% of cases, respectively, which are then classified, in turn, as active or inactive forms based on their inflammatory status [18,20]. A study by Acquaviva and coworkers demonstrated that peripheral blood mononuclear cell (PBMC) transcriptomes contain useful information for classifying the different subtypes of MS [21].

There is still an incomplete understanding of pathomechanisms of MS both at onset and in the following stages, suggesting a critical unmet need for prognostic and diagnostic biomarkers. It is no coincidence that the diagnosis is based, still now, on physiopathological criteria of dissemination in time and space of the CNS lesions, in the absence of a known etiology.

Recently, EVs and their bioactive cargo have been emerging tools with great diagnostic and prognostic potential in the realm of precision medicine for a wide type of diseases, including those of the CNS. Here, we assessed if electronic microscopy analysis of ultrastructural alterations in PBMCs from MS patients coupled with the study of EV cargo isolated from plasma can be considered a suitable approach for biomarker discovery in MS.

This is a pilot study on the ultramicroscopic assessment of PBMCs and EVs in MS patients. The results can have a cross-sectional and a real-world cohort characterization, due to the type of data observation and collection. Although the limited sample size hampers generalization onto a huge population, the study reached its primary endpoint in finding detectable differences between HCs and cases.

Peripheral immune cells have been proposed as key players in MS, as several immunosuppressant drugs targeting T- and B-lymphocytes have demonstrated a beneficial effect on RRMS patients [22,23]. Furthermore, monocytes have been proposed to be involved in MS pathogenesis and/or disease severity [24] even if their intimate role remains elusive.

In the MS paradigm, activated lymphocytes and monocytes can reach the CNS *en masse*, mainly via structural alteration of BBB or across the choroid plexus, and are decisively involved in inflammatory lesion development. Crosstalk between cells is pivotal for triggering and propagating immune cell responses. Even if soluble factors, including chemokines and cytokines, are widely accepted as the main drivers in neuroinflammation [25], EVs are gaining increased attention due to their characteristic of transferring immunomodulatory mediators between cells throughout the BBB, from the CNS to the systemic circulation or vice versa [26]. Thus, peripheral immune cells, CNS resident cells, and endothelial cells are no longer considered bystanders, but fully involved elements in MS pathophysiology [1,2,3].

Our observations suggest that differences between healthy controls and MS patients exist in terms of the ultrastructural shape of PBMCs and plasma-derived vesicle numbers and content. Obviously, these findings refer to RRMS in the inactive phase, as suggested by the relapse-free state of the enrolled patients.

In MS patients, the number of activated PBMCs increases as well as the number of multivesicular bodies (MVBs) containing vesicles that are released from cells as single vesicles or as vesicles contained within membrane-surrounded structures. These structures could be a specific type of EV, known as migrasomes. The latter are released by migrating cells, having an important role in various intercellular communication processes and substance transfer [27].

We also observed a high number of endosomal vesicles in samples from diseased subjects as well as plasma-membrane-covered extensions, with multiple large vesicles and numerous autophagosomal vacuoles containing undigested cytoplasmic material. These morphostructural shape changes are typical of activated cells and compatible with a condition of altered membrane trafficking machinery of stressed cells living in the inflammatory environment [28], such as in MS. The undigested content of the autophagosomal vacuoles is consistent with the postulated protein maturation defect.

Furthermore, amorphous material was observed around the bigger vesicles present on plasma membrane extensions, similar to a glycocalyx. Its thickness and composition are known to be involved in several physiological and pathological functions, including the regulation of membrane protein diffusion [29] and vesiculation [30]. This is confirmed by transmission electron microscopy analysis of vesicles isolated by the plasma of patients, i.e., only bigger vesicles show electron-dense material around the membrane. Furthermore, two different vesicle populations with an average diameter of 87 nm and 240 nm were observed, whose percentage of abundance was 84% and 16%, respectively. Based on the results obtained by transmission electron microscopy and previous results achieved [14,31,32] regarding some key regulatory molecules, we decided to analyze the cargo of EVs isolated from plasma of MS patients using Western blot analysis. To this end, we chose to evaluate GANAB, TGFβRII, NF-κB, Cortactin, Septin 2, IFI35, ARHGDIA, and Cofilin 1 as molecules involved in different molecular processes of MS. First, we noted that the expression of all of these molecules in sEVs was always higher in cases compared to controls. Interestingly, ARHGDIA and Cofilin 1 were only expressed in this compartment, not in *l*EVs. Considering the functional role of the studied proteins and the endosomal endocytic biogenesis of sEVs [33], this finding suggests an inflammatory dysregulation in membrane trafficking, resulting in the proinflammatory release of small vesicles on the target organ. Secondly, we noted significant differences between MS patients and HCs in the protein expression of both large and small vesicles.

GANAB, heterodimeric enzyme α-glucosidase II, is a member of the glycosyl hydrolase 31 (GH31) family of proteins, located in the endoplasmic reticulum and devoted to hydrolyzing the three terminal glucoses on the N-linked oligosaccharide during the *N* glycosylation mechanism, linked to the protein maturation. A significant correlation between GANAB expression and MS was evidenced in RRMS patients undergoing IFN therapy by using Western blotting from PBMCs and MRI post-analysis of the brain. In particular, GANAB can be associated with the disease progression phase and, thus, GANAB could be considered as a biomolecular marker of neuroinflammation and treatment response in MS [14,34]. In fact, the steps (adhesion, tethering, and rolling on BBB blood vessels) supporting the process of invasion of lymphocytes in the CNS are mediated by glycoproteins in MS [35,36].

In MS patients, this enzyme is already known to undergo dysregulation. In our observations, GANAB was expressed in both sEV and *l*EV compartments, confirming its endosomal subcellular localization, but also in the plasmalemma-derived vesicles, depending on the functional state of cells in the context of disease.

Cytoplasm-localized interferon-induced protein 35 (IFI35) is considered to be a damage-associated molecular pattern (DAMP) involved in the elicitation of innate immunity and inflammatory exacerbation by Toll-like receptors (TLR) [37]. IFI35 is differentially expressed in IFN-treated MS patients compared to untreated MS patients and healthy controls, and can also be indicated as a marker of neuroinflammation in MS [31,34,38]. Interestingly, this molecule is expressed in the sEVs of most patients, but not in healthy controls. On the contrary, IFI35 is expressed in all *l*EV samples, with a variable ADU, ranging from normal to slight upregulation. In fact, the molecule regulates targeted cells in a proinflammatory manner, suggesting both an endosome and microvesicular pattern.

The actin-binding protein, Cortactin, is involved in various cellular processes strictly dependent on membrane dynamics and cell motility, including cell adhesion, migration, and endocytosis [39,40]. In particular, its expression in endothelial cells represents a key event in the control of the endothelial barrier integrity and in supporting diapedesis as well as the consequent inflammation process [41,42,43,44]. In 2020, Samus demonstrated that cortactin contributes to the development of neural inflammation by supporting leukocyte transmigration in the CNS through the BBB. In fact, cortactin gene inactivation in endothelial cells counteracts the entry of CD4+ and myeloid cells into the CNS and, consequently, inflammation and demyelination decrease as well as symptoms in EAE, the in vivo model of MS [45]. In our observations, Cortactin is variably more expressed in sEVs than in *l*EVs. In fact, this factor is involved in the endosome biogenesis, which is, in turn, enhanced in the inflammatory process.

Septin 2 is a member of the septin family proteins, which has important roles in several membrane-associated processes, including regulation of cell morphology, mitochondrial fission, endomembrane fusion, and migration, by interacting with actin filaments and microtubules [46]. Moreover, Septin 2 is localized at the junctions of endothelial cells, and it is required to preserve the proper organization of the microvascular endothelial cell monolayers. It has been demonstrated that during inflammation, the increase of TNF-α causes increased permeability of the endothelium with partial loss of cell junction integrity by impacting Septin 2 functionality [47] and with, in turn, the possibility that immune cells reach the CNS and exacerbate inflammation. Upregulated Septin 2 denotes a generalized high vesicular fusion activity in MS patients, in both endosomal and EV compartments. Septins also contribute to controlling vesicular trafficking machinery at several steps, including the SNARE-mediated membrane fusion and putative blood brain barrier (BBB) permeability. The BBB plays a crucial role in demyelinating diseases of the CNS. In fact, the BBB breakdown precedes the nervous tissue invasion and lymphocyte reactivation before myelin damage and axonal injury. Moreover, calcium entry into the ER is inhibited by septins, contributing to ER stress regulation [48]. ER stress has recently been postulated as another biological process in MS pathology.

We found Septin 2 as a variably expressed factor in EVs, from the absence to the upregulation, depending on the functional state of the cell in the systemic context of neuroinflammation. This molecule works on the plasmalemma surface as well as in the endosomal compartment, depending on the endocytosis or multivesicular body formation. The latter process is known to take place during inflammation.

Cofilin is an actin-binding protein expressed in two isoforms: Cofilin 1 is present in non-muscle tissues and Cofilin 2 is the major isoform in differentiated muscle. The main role of Cofilin is the regulation of actin dynamics and related processes such as cytoskeletal polymerization. In the CNS, Cofilin 1 is involved in the motility and guidance of the neuronal growth cone, dendritic spine organization, axonal branching, and synaptic signaling.

Recently, it has been suggested that Cofilin could be involved in apoptosis induction, mitochondrial dynamics, microtubule instability, and the regulation of gene expression [49]. Alterations in these processes are involved in many inflammatory diseases of the CNS, but their role remains to be elucidated [50]. The isoform Cofilin 2 increases in the serum of Alzheimer’s disease patients, and it has been suggested as a highly sensitive and specific diagnostic biomarker [51]. In our observations this factor is represented only in sEVs, with an expression similar to that of controls. Consistently, Cofilin 1 is believed to inhibit the movements of mitochondria and endosomes, resulting in a synaptic loss process, the main mechanism of neurodegeneration and brain aging [52].

ARHGDIA (Rho GDP Dissociation Inhibitor Alpha) is a protein involved in the Rho GTPases-dependent signaling processes. Diseases associated with ARHGDIA include Nephrotic Syndrome, which can also be caused in MS patients by IFNβ-1a treatment, used to counteract neuroinflammation [53]. Like Cofilin, this factor was represented only in sEVs, except in controls and patient 2. This is consistent with its endomembrane localization and in the Golgi apparatus (not in the plasmalemma, as previously thought). This localization promotes other inflammatory-related phenomena, such as exocytosis and the formation of immunological synapses.

Moreover, TGFβRII from sEVs was generally upregulated in our MS patients compared to the HCs. This factor underwent the opposite regulation in *l*EVs, apart from in patient 3. These observations are consistent with the role of TGFβ in endocytosis and intercellular communication, as well as signal transduction through the clathrin and SMAD pathways [54].

Likewise, NF-κB from diseased subjects was upregulated in our sEV cohorts. On the contrary, it was variably expressed in *l*EVs. This factor works in two ways in inflammation, depending on the TLR and the endosome pathway.

Considered together, these data evidence, in the diseased subjects, a biological process based on molecule events and vesicle dynamics. In other words, this biological process involves a cellular as well as a humoral component, such as the mononuclear elements of the peripheral blood and several molecules as the driving force of inflammation. The latter is denoted just by the quality of the target molecules, already known as pro-inflammatory in nature, including IFI35, NF-κB, and GANAB. Peripheral lymphomonocytes are thought to be associated with immune-mediated responses, in particular the adaptive response, resulting in the activated ultrastructural shape.

Our data evidence the absence of Cofilin 1 and ARHGDIA in the *l*EV content, denoting residual cytokinesis and low signal transduction at the plasmalemma interface. Similarly, high levels of these factors in the sEV compartment correlate with elevated cytoskeletal depolymerization in the origin cells, as well as a high activity in the second messenger system. Thus, despite the inflammatory content of sEVs denoted by IFI35, NF-κB, and GANAB, the cytokinetic machinery is residual, unlike the vesiculation machinery.

Due to the small sample size, we cannot now attribute to these observations the dignity of a molecular pattern of MS, much less can we postulate a clinically putative relevance to them. However, we can state that this methodology improves our cognitive horizons for the disease. This has already occurred in ALS. In this field, the study of vesicle content evidenced a number of molecules serving as inflammatory signals in the cell-to-cell communication that had been unknown in the recent past, resulting in novel study matter. In MS, preceding studies have evidenced a reduction in the number of the EVs as a result of the DMTs, confirming our finding of their inflammatory significance [55,56].

In summary, our study evidenced the electronic microscopy of PBMCs coupled with Western blot on EV content as a suitable study method in MS. With this methodology we showed altered vesicular dynamics and morphological shapes of cell activation in the peripheral lymphomonocytes from MS patients.

Moreover, we showed an extracellular vesicular content electively oriented in remotely transducing an inflammatory signal as well as in reflecting the inflammatory condition of the origin cell in MS. Due to the limited sample size, statistical correlation between these findings and other parameters is difficult. However, our results reflect the experimental conditions, that is, the relapsing remitting form of MS, its quiescent phase, and the “dissemination in space” of the lesion load, according to McDonald international guidelines [57]. On the other hand, the applied method targeted the morphological and molecular differences between the compared cohorts, resulting in a reliable tool of investigation, which, indeed, was the goal of the study.

## 4. Materials and Methods

### 4.1. Study Design

In a case-control, cross-sectional, ultramicroscopic, and proteomic study, we enrolled 4 RR untreated MS patients, afferent to the Multiple Sclerosis Centre of Neurological Department at the “F. Ferrari” Hospital in Casarano, Lecce (Italy), and 4 healthy controls (HCs). All subjects were matched for sex, age, and ethnicity. Each enrolled subject underwent blood withdrawal after enrolment and PBMCs were extracted. In addition, the patients underwent brain and spinal cord MRIs to exclude co-existing relapse. The MRI images (including T1w, T2w, FLAIR, 3DT1, 3DFLAIR) closest to time withdrawal were used for the assessment of the disease activity in each patient, using gadolinium as paramagnetic contrast. EM and Western blotting analysis of EV content isolated from venous blood of MS patients were performed at Di.S.Te.B.A. at the University of Salento, Lecce (Italy). In addition, for each enrolled MS subject, data were collected on disease duration (DD) and age at onset (AO).

### 4.2. Study Population

Based on adequate information, the subjects’ enrolment took place at the MS Centre of Casarano during routine visits, with reference only to inclusion/exclusion criteria.

*Inclusion criteria*: RRMS untreated patients. All MS patients were previously diagnosed according to the 2017 McDonald criteria [57] and imaged in an exacerbation-free period of at least three months. All enrolled subjects expressed written informed consent for enrolment in the study. The study was conducted in accordance with the Declaration of Helsinki and the protocol n. 1210/DS, and was approved by the Local Ethics Committee of A.S.L.LE.

*Exclusion criteria*: any metabolic, cardiovascular, or immunological comorbidity (cardiovascular, immunological, and metabolic, such as atheromasia or prior strokes, diabetes, arthritis, rheumatoid arthritis, and connective tissue disorders); local or systemic transient inflammatory and septic conditions (bacterial infections, cold, cough, flu, exanthematous diseases and viruses, relevant trauma, smoking, obesity); finally, any non-MS treatment in the previous three months as well as all patients expressing abnormal cell count at the hemocromocytometric examination before the study entry. These criteria exclude the subjects having possible confounding factors, such as metabolic and local or systemic inflammatory processes.

### 4.3. MRI Protocol

Standard MRI of the MS patients was performed on a 1.5-T Philips MR apparatus (180 mT/m) (Achieva, Philips Medical Systems, Best, The Netherlands) in accordance with international guidelines [58]. The acquisition sequence types were SE T1–TSE T1 MT–BRAIN VIEW FLAIR 3-D; acquisition time 2.170–3.070–4.140; field of view 230 3183 mm AX–250 3250 FLAIR SAG–180–200 3180 mm COR MT; orientation: TRA–COR–TRA; alignment: TRA–COR–TRA; and voxel size: 0.89/0.88/4–0.56/0.56/4–0.31/0.31/0.6, respectively. Repetition time (TR) was 450–614–4800; echo time (TE) was 15–12–307; and inversion time (TI) was −/−/1660. The flip angle was 69°–90°−/ and the NEX was 1–2–2. SENSE parallel imaging method and contrast enhancement (Gadovist single dose, 10 min post administration) were used. Axial images (ax) were acquired from all T1- and T2-weighted sequences; axial and three-dimensional images (3DFLAIR) were acquired from FLAIR sequences. Among the image transferring systems, 3DSlicer (3DSlicer v.5.6.2.32448) and OsiriX (OsiriX 12.0 Release) software were used as DICOM nodes to manage the resonance images once acquired and sent from the NMR apparatus. Thus, the pre- and post-contrast standard image examinations of the brain and spinal cord were obtained.

### 4.4. PBMC Isolation

Sixteen mL of heparinized venous blood was diluted with PBS (Phosphate Buffered Saline) in a 1:1 solution and, after platelet-rich plasma discharge, it was layered on Ficoll-Hypaque density gradient (GE Healthcare, Hertfordshire, UK). Thus, the PBMC ring was obtained by centrifugation (800× *g* for 20 min, acceleration 2, brake 0) and the cells were manually aspirated and washed twice in the isotonic PBS solution. Finally, PBMCs were resuspended for 15 min at room temperature in 1 mL of double distilled water to obtain the lysis of the contaminant erythrocytes, with a final purity of 93–97% [59].

### 4.5. EV Isolation

Five mL of plasma was subjected to differential centrifugation using a Beckman Coulter Ultracentrifuge Optima XE (Beckman Coulter, Brea, CA, USA). Briefly, the plasma was centrifuged successively at 500× *g* (10 min, room temperature (RT)), 800× *g* (10 min, RT), 2000× *g* (20 min, RT) to remove aggregates. Then, the resulting supernatant was centrifuged at 20,000× *g* (20 min, 4 °C). The large vesicle-enriched pellets (*l*EVs) were collected, and the supernatant was filtered through 0.22 µm filters (polyethersulfone filter units, Thermo Fisher Scientific, Waltham, MA, USA). The filtered supernatant was centrifuged at 100,000× *g* (70 min, 4 °C) to collect the small vesicle-enriched pellets (sEVs) [60].

### 4.6. EV Characterization

*Transmission electron microscopy (TEM)*: 3–5 µL of each EV sample suspended in PBS was put on a carbon-coated grid. The grids were observed with a TEM Hitachi 7700 (Hitachi High Technologies America Inc., Dallas, TX, USA) operating at 100 kV.

*Western blotting analysis*: sEVs and *l*EVs were analyzed for the protein expression of specific markers of EVs (Annexin I, Alix, Calnexin, CD63, Tsg101, Flotilin, and HSP90) as detailed below. Furthermore, the cargo of vesicles was analyzed by considering the following molecules: GANAB, TGFβRII, NF-κB, Cortactin, Septin 2, IFI35, ARHGDIA, and Cofilin 1.

EVs were lysed in ice-cold radioimmunoprecipitation assay (RIPA) buffer (Thermo Fisher Scientific, Waltham, MA, USA) in agitation for 30 min. Insoluble material was pelleted by centrifugation for 30 min at 13,000× *g* at 4 °C. Supernatants were transferred to a new tube, and the protein concentration was measured by Bradford assay (Biorad, Hercules, CA, USA). Proteins (10 μg) were separated by sodium dodecyl sulfate-polyacrylamide gel electrophoresis (10–12% polyacrylamide, SureCast Acrylamide Solution, Thermo Fisher Scientific, Waltham, MA, USA) and transferred to nitrocellulose membranes. The membranes were blocked with 5% non-fat dry milk in Tris-buffered saline containing 0.1% Tween 20 (TTBS) for 1 h at room temperature. Then, the membranes were incubated overnight at 4° C with following primary antibodies: anti-CD63 (#365604 monoclonal Ab, 1:500 dilution; Santa Cruz Biotechnology, Dallas, TX, USA), anti-Annexin A1 (#sc-130305 monoclonal Ab, 1:500 dilution; Santa Cruz Biotechnology, Dallas, TX, USA), anti-Alix (#sc-53540 monoclonal Ab, 1:500 dilution; Santa Cruz Biotechnology, Dallas, TX, USA), anti-Calnexin (#sc-23954 monoclonal Ab, 1:500, Santa Cruz Biotechnology, Dallas, TX, USA), anti-TSG101 (#sc-7964 monoclonal Ab, 1:500 dilution; Santa Cruz Biotechnology, Dallas, TX, USA), anti-Flotilin (#sc-74566 monoclonal Ab, 1:500 dilution; Santa Cruz Biotechnology, Dallas, TX, USA), anti-HSP90 (#H1775 monoclonal Ab, 1:1000, Sigma-Aldrich, St. Louis, MO, USA), anti-GANAB (#SAB1401584 polyclonal Ab, 1:1000, Sigma-Aldrich, St. Louis, MO, USA), anti-IFI35 (#WH0003430M1 monoclonal Ab, 1:1000, Sigma-Aldrich, St. Louis, MO, USA), anti-Septin 2 (#SAB1406173 polyclonal Ab, 1:1000, Sigma-Aldrich, St. Louis, MO, USA), anti-ARHGDIA (#SAB1405483 polyclonal Ab, 1:1000, Sigma-Aldrich, St. Louis, MO, USA), anti-Cortactin (#SAB1305513 monoclonal Ab, 1:1000, Sigma-Aldrich, St. Louis, MO, USA), anti-Cofilin 1 (#SAB2702206 monoclonal Ab, 1:1000, Sigma-Aldrich, St. Louis, MO, USA), anti-TGFβRII (#sc-17792 monoclonal Ab, 1:2000 dilution; Proteintech Europe, Manchester, UK), and anti-NF-κB (#sc-372 polyclonal Ab, 1:500 dilution; Santa Cruz Biotechnology, Dallas, TX, USA), diluted in TTBS in 5% non-fat dry milk. After washing with TTBS, the membranes were incubated for 1 h at room temperature with the following horseradish peroxidase-conjugated secondary antibody diluted in TTBS in 5% non-fat dry milk: rabbit anti-mouse IgG (#G-21040 polyclonal Ab, 1:5000 dilution; Invitrogen, Waltham, MA, USA), goat anti-rabbit IgG (#G-21234 polyclonal Ab, 1:5000 dilution; Invitrogen). The immunoreactive bands were detected by ChemiDoc Imaging System (Bio-Rad Laboratories, Hercules, CA, USA) using a commercial enhanced chemiluminescence (ECL) reagent (Immobilon Crescendo Western HRP substrate; Merck Millipore, Darmstadt, Germany). The density of the specific bands was quantified by densitometric analysis performed by ImageJ (ImageJ 1.53e, Wayne Rasband and contributors, National Institutes of Health, USA). The relative density of each analyzed protein was calculated by dividing each peak area by the corresponding one from the control sample. Contrastingly, the HC well was loaded with a sample from a pool of the four controls. This solution provides a more useful graph without altering the statistical reliability.

### 4.7. TEM Protocol

PBMCs were fixed with 2.5% glutaraldehyde in 0.1 mol/L cacodylate buffer pH 7.4 for 1 h at a freezing temperature and post-fixed with 1% OsO4 in the same buffer for 2 h at a freezing temperature. Then, cells were stained with a solution of uranyl acetate (5% in water) overnight. Cells were then dehydrated with increasing degrees of ethanol (25%, 50%, 70%, 90%, and 100%) and embedded in Epoxy Spurr resin (#4221D-1; TAAB Laboratories Equipment Ltd., Aldermaston, UK). The 60 nm sections were examined under a Hitachi 7700 (Hitachi High Technologies America Inc., Dallas, TX, USA) transmission electron microscope (TEM) operating at 100 kV.

### 4.8. Statistical Analysis

All data are expressed as mean ± SD. Difference between means was performed using the non-parametric Mann–Whitney test. Multiple comparisons between two groups were performed by using the non-parametric Kruskal–Wallis test, with the Dunn’s correction test applied, and *p* < 0.05 was considered to be significant (GraphPad Prism 9 software, GraphPad Software, San Diego, CA, USA).

## 5. Conclusions

In conclusion, the electronic microscopy of PBMCs coupled with plasma Western blot analysis applied to MS revealed lymphomonocytes as non-resting cells, but morphologically characterized activated elements and the EVs as biological transducers belonging to the inflammatory signaling pathway, with a putative pathological relevance in the relapse-free disease phase. This is an innovative methodology representing a potential improvement in our cognitive horizons and it also represents an emerging study tool in the MS field, with clinical applications that should be explored soon.

## Figures and Tables

**Table 1:** The arbitrary densitometric unit percentage (ADU) of cargo in large (*l*EVs) and small (sEVs) vesicles isolated from the plasma of healthy control (HC) and MS patients (P1–P4).

	sEVs					*l*EVs				
	*HC*	*P1*	*P2*	*P3*	*P4*	*HC*	*P1*	*P2*	*P3*	*P4*
**GANAB**	5.51 (±0.28)	36.58 (±1.6)	10.04 (±0.47)	11.62 (±0.5)	36.26 (±1.77)	17.14 (±0.86)	18.74 (±0.98)	2.81 (±0.18)	16.07 (±0.73)	45.24 (±1.99)
**TGFβRII**	6.91 (±0.27)	29.10 (±1.44)	26.97 (±1.36)	20.49 (±0.98)	16.54 (±0.79)	36.62 (±1.93)	N.D.	0.35 (±0.01)	55.96 (±2.8)	7.06 (±0.29)
**NF-κB**	9.57 (±0.47)	23.29 (±1.1)	24.10 (±1.2)	21.13 (±0.99)	21.90 (±1.05)	28.60 (±1.39)	0.88 (±0.04)	25.66 (±1.3)	37.18 (±1.91)	7.67 (±0.38)
**Cortactin**	0.54 (±0.03)	8.13 (±0.39)	21.17 (±1.01)	19.41 (±1.01)	51.24 (±2.6)	33.50 (±1.77)	11.06 (±0.53)	12.15 (±0.56)	37.19 (±1.85)	6.1 (±0.28)
**Septin 2**	7.13 (±0.36)	18.4 (±0.94)	33.17 (±1.53)	22.36 (±1.23)	18.94 (±0.97)	22.38 (±0.99)	N.D.	23.22 (±1.13)	38.23 (±1.91)	16.18 (±0.76)
**IFI35**	0.04 (±0.01)	27.76 (±1.38)	0.05 (±0.01)	59.81 (±2.88)	12.34 (±0.77)	14.79 (±0.7)	14.17 (±0.72)	26.98 (±1.32)	29.2 (±1.52)	14.86 (±0.7)
**ARHGDIA**	N.D.	12.41 (±0.65)	N.D.	57.92 (±2.76)	40.84 (±2.1)	N.D.	N.D.	N.D.	N.D.	N.D.
**Cofilin 1**	17.95 (±0.87)	24.01 (±1.1)	14.74 (±0.77)	20.81 (±0.96)	22.49 (±1.15)	N.D.	N.D.	N.D.	N.D.	N.D.

## Data Availability

The original contributions presented in the study are included in the article, further inquiries can be directed to the corresponding authors.
